# Near-Infrared Fluorescent Agent for *In Vitro* Screening of Endometrial Cancer and Precancerous Lesions

**DOI:** 10.3389/fonc.2021.713583

**Published:** 2021-07-01

**Authors:** Dongxin Liang, Xiaoqian Tuo, Qing Wang, Lanbo Zhao, Kailu Zhang, Yiran Wang, Xue Feng, Panyue Yin, Lin Guo, Yu Liu, Lei Wang, Lu Han, Ruifang An, Qiling Li

**Affiliations:** ^1^ Department of Obstetrics and Gynecology, First Affiliated Hospital, Xi’an Jiaotong University, Xi’an, China; ^2^ Department of Pathology, First Affiliated Hospital, Xi’an Jiaotong University, Xi’an, China

**Keywords:** endometrial cancer, FR-α, *in vitro* diagnosis, near-infrared targeting fluorescent dye, ZW-FA

## Abstract

The lack of cytopathologists delays the advancement of screening for endometrial cancer. It was urgent to develop a new dye for rapid diagnosis. Our study aimed to synthesize a targeted folate receptor-α near-infrared (NIR) fluorescent agent, folic acid-zwitterionic NIR fluorophore (ZW-FA), and explore the feasibility for screening of endometrial cancer and precancerous change. Folic acid was conjugated with zwitterionic NIR fluorophore. The preparation of ZW-FA was validated by ^1^H NMR, mass spectrometric, ultraviolet spectra and fluorescence spectra. ZW-FA was incubated with endometrial cytology samples obtained from patients who underwent dilation and curettage or total hysterectomy. Diagnostic utility was calculated by applying laser confocal microscope, Image-J and statistical models, such as enumeration, receiver operating characteristic curve, logistic regression, support vector machine and decision tree were used. The purity of ZW-FA was > 95% determined by ^1^H NMR. ZW-FA had the strongest absorption peak at 633 nm in ultraviolet spectra. Photostability of ZW-FA was over 8 hours. In clinical validation, a total of 92 patients were enrolled. The cut-off value of ZW-FA was 49 in enumeration, which was used to distinguish the type of samples. Indicators about diagnostic utility are as follows: sensitivity 90.77%, specificity 62.96%, false-positive rate 37.04%, false-negative rate 9.23%, positive predictive value 85.51% and negative predictive value 73.91%. The samples processed by ZW-FA did not affect further Hematoxylin-Eosin staining and pathological diagnosis. It was an effective cytologic strategy for *in vitro* diagnosis of endometrial cancer and precancerous change by using ZW-FA.

**Clinical Trial Registration:**

http://www.chictr.org.cn/index.aspx, identifier ChiCTR1800020123.

## Introduction

Endometrial cancer is the most common malignancy of the female reproductive system in developed countries (1). It is urgent to carry out screening of endometrial cancer and precancerous change (2). Endometrial cytology is the most common test for the initial evaluation of endometrium in some countries (3). While the extreme lack of cytopathologists delays the advancement of screening.

Near-infrared (NIR) fluorescence imaging has emerged as a non-invasive and real-time visualisation technique. Compared to conventional fluorescent dyes, NIR dyes show ultralow autofluorescence, providing high signal-to-background ratio images (4). Zwitterionic NIR fluorophore (ZW800-1) has the characteristics of high hydrophilicity, stable structure, charge balance and ultra-low non-specific tissue uptake (5). ZW800-1 was labelled on the interferon-α molecule of the hyaluronate-interferon α for targeted therapy of the hepatitis C virus (6). Combining ZW800-1 with cyclic arginine-glycine-aspartate (RGD) peptide, scholars have proved that the cRGD-ZW800-1 provides clearer identification in colorectal cancer (7).

Folate receptor-α (FR-α) is a 38 to 40 kDa molecule with a high affinity for folic acid and its derivatives (8). FR-α in normal tissue is restricted to apical surfaces of some organs such as kidney, lung and choroid plexus (9). Overexpression of FR-α has been reported in various solid tumours such as endometrial cancer, ovarian cancer, breast carcinoma, non–small cell lung cancer and so on (10–13). FR-α is expressed in 41%-89% of endometrial cancers and is highly expressed in 40% of endometrial cancers. It was reported that FR-α is expressed in 63%-81.1% of endometrioid cancers, 82% of serous cancers, and 83% of clear cell cancers. High expression of FR-α was found in 12%-50.5% of endometrioid cancers, 33%-41% of serous cancers, and 25% of clear cell cancers (14–17). Based on the different expression of FR-α, antibody drugs targeting FR-α have been developed previously (10, 18–22). Results of a combination of folic acid and high-density lipoprotein fluorescent nanoparticles showed that with the linking of folic acid, the uptake of fluorescent nanoparticles in ovarian cancer tissues significantly increased, which is an excellent targeted drug (23). The coupling of folic acid and indocyanine green demonstrated that folic acid increased the absorption of the complex by breast cancer cell lines MCF-7, which are of FR-α overexpression (24). In summary, the coupling with folic acid improved the tumor targeting of the compound.

Nevertheless, related agents used in the diagnosis of endometrial cancer have rarely been developed yet. In this study, we conjugated folic acid and ZW800-1 to construct a targeted FR-α near-infrared fluorescent agent folic acid-ZW800-1 (ZW-FA) (patent number: ZL201510104185.5). After completing the synthesis and characterization of ZW-FA, a cytologic test for *in vitro* diagnosis of endometrial cancer and precancerous change was proposed to verify the feasibility.

## Materials and Methods

### Synthesis of the ZW-FA Compound

The synthesis of ZW-FA was composed of eight steps. In this part, 3-methyl-2-butanone and 4-hydrazinobenzenesulfonic acid were purchased from Aladdin, 4-(2-carboxyethyl) phenylboronic acid and 1,1,3,3-tetramethoxypropane were purchased from Alfa Aesar and 3-Bromo-N,N,N-trimethylpropan-1-aminium bromide was purchased from Ark Pharm. All solvents and other reagents were of reagent grade quality and purchased commercially.

### Characterization

The preparation of ZW-FA was validated by ^1^H NMR, mass spectrometric, ultraviolet spectra and fluorescence spectra. ^1^H NMR spectra were recorded on Varian unity INOVA-400 spectrometer at 400, taking TMS as an internal standard. Mass spectrometric detection was performed on a spectrometer (microTOF-Q II ESI-Q-TOF LC/MS/MS, Bruker Daltonic Inc., USA). Ultraviolet spectra were recorded using ultraviolet spectrophotometer (UV/VIS spectrophotometer UV-2700, Shimadzu, Ltd., Japan). Fluorescence spectra were recorded using a luminescence spectrometer (F-7100, Hitachi, Ltd., Japan). Photostability of ZW-FA (7.14 ug/ml) was evaluated in a variety of biological media, including H_2_O, phosphate-buffered saline (PBS), serum and blood at 37°C under continuous laser exposure at 650-850 nm (Cary series UV-Vis; Agilent Technologies Co., Ltd., USA).

### Fluorescence Imaging of Cell Lines

Human ovarian cancer cell line SKOV_3_ and human breast cancer cell line MDA-MB-231 with overexpression of FR-α were conducted as positive cell lines and obtained from the Cell Bank of the Chinese Academy of Sciences (Shanghai, China). Human umbilical vein endothelial cell line HUVEC was performed as a negative cell line and obtained from the American type culture collection in the United States. All cell lines were grown in complete medium without folic acid and maintained under humid conditions with 5% CO_2_ at 37°C.

All cells were cultured on slides in a 24-well plate at 10^5^/well overnight. Each slide was washed 3 times with PBS, and the cells were fixed with 0.5 ml of 4% paraformaldehyde for 30 minutes and washed with PBS again.

To verify the targeting of ZW-FA to FR-α, a monoclonal antibody validation experiment was performed. SKOV_3_ was incubated with human folate receptor alpha-1 (FOLR1) antibody (catalogue #MAB5646, R&D, USA) with 10 µg/ml and Alexa Fluor 488 (catalogue #ab150117, Abcam, Australia). Then the cell was incubated with ZW-FA at 600 ug/ml for 1 hour at 37°C, washed again with PBS and incubated with DAPI (catalogue # 10236276011, Roche, Switzerland) for 30 minutes.

Similarly, in concentration-dependent experiment, SKOV_3_ was incubated with ZW-FA at 400 ug/ml, 500 ug/ml and 600 ug/ml for 1 hour at 37°C. MDA-MB-231 and HUVEC were incubated with ZW-FA at 600 ug/ml for 1 hour at 37°C. They were washed again with PBS and incubated with DAPI for 30 minutes.

Finally, antifade mounting medium (catalogue #P0126, Beyotime, China) was used for sealing, and laser confocal microscope (C2, Nikon, Japan) was used to observe the results. The excitation wavelengths of DAPI, human FOLR1 antibody and ZW-FA fluorescence were 408 nm, 488 nm and 633 nm respectively. It’s worth noting that the incubation and afterward steps needed to be protected from light.

### Patients Enrolled

Endometrial cytological samples of enrolled patients had been collected by Li Brush (20152660054, Xi’an Meijiajia Medical Technology Co. Ltd., China) from patients who had undergone dilation and curettage (D&C) or total hysterectomy during 15 months (07/2018 to 10/2019) (25–28). The detailed clinical information of enrolled patients had been collected from Electronic medical record system.

This study was performed in accordance with the Declaration of Helsinki and approved by the Ethics Committee of the First Affiliated Hospital of Xi’an Jiaotong University (XJTU1AF2017LSK-100) and informed written consent was obtained from all patients before the study.

### Sample Processing

The samples were stained with ZW-FA and Hematoxylin-Eosin (H&E) successively. The slides loaded with endometrial cells were fixed in 95% alcohol for 30 minutes and washed with PBS (25). We incubated the cytology slides with ZW-FA (500 ug/ml) for 1 hour at 37°C, washed with PBS and incubated with DAPI for 30 minutes at last. Laser confocal microscope was used to observe results. The excitation wavelengths of DAPI and ZW-FA fluorescence were 408 nm and 633 nm respectively. It’s worth noting that the steps of incubation and afterward needed to be protected from light.

After data collection, H&E staining and cytopathological diagnosis were performed by two experienced pathologists to verify whether ZW-FA staining affected subsequent H&E staining (25). For samples with inconsistent diagnosis results, a third pathologist re-diagnosed.

To evaluate the diagnostic utility of ZW-FA, postoperative histopathologic results were regarded as the gold standard (**Figures 4D, H**). The histopathologic diagnoses, according to the International Society of Gynecological Pathology Classification, included endometrial carcinoma, endometrial atypical hyperplasia, complex hyperplasia defined as adenomatous hyperplasia without atypia, mixed endometrium, simple hyperplasia including cystic glandular hyperplasia, atrophic endometrium, proliferative endometrium and secretory endometrium (29). The cytopathologic diagnoses were classified as follows: endometrial cancer cells, endometrial atypical cells, endometrial hyperplasia cells, mixed endometrial cells, atrophic endometrial cells, proliferative endometrial cells and secretory endometrial cells (27). Positive results were defined as endometrial carcinoma, endometrial atypical hyperplasia in histopathology and endometrial cancer cells, and endometrial atypical cells in cytopathology. Other diagnoses were defined as negative.

### Immunocytochemistry

The false-negative and false-positive samples of ZW-FA were subjected to immunocytochemistry (ICC) to identify whether FR-α was expressed by using human folate receptor alpha-1 (FOLR1) antibody (catalogue #MAB5646, R&D, USA) with 20 µg/ml (30).

The staining intensity of FR-α was divided into 0, no staining; 1, weak; 2, moderate and 3, strong, as described by Henry et al. (31). Evaluation of ICC staining was performed independently by two experienced experts, for samples with inconsistent results, a third observer re-evaluated.

### Data Analysis

Fluorescence images of each sample were collected by laser confocal microscope. Image-J (Image-J 1.52a) was used to analyze all images and obtain the grey values of them. Then statistical prediction models were built to analyze the above grey values. Statistical prediction models included receiver operating characteristic curve (ROC), enumeration (ENUM), logistic regression, support vector machine (SVM) and decision tree. The ROC curve was used to obtain the cut-off value to calculate the diagnostic utility. The diagnostic utility corresponding to each gray values were listed in ENUM method, and the most appropriate cut-off value would be obtained by comparing all the results. Logical regression model was established to predict the positive probability of each gray value, and then the ROC curve was drawn to calculate the diagnostic utility. The SVM model was used to analyze the data from training set to distinguish the positive and negative in the test set. The cut-off value was calculated by using the decision tree model and then the diagnostic utility was calculated.

According to the models, all cytological samples were divided into positive and negative. Indicators about diagnostic utility of ZW-FA were calculated through the confusion matrix for evaluating the feasibility in endometrial cytology *in vitro* diagnosis. Indicators were as follows: sensitivity (Se), specificity (Sp), false-positive rate (FPR), false-negative rate (FNR), positive predictive value (PV+) and negative predictive value (PV–). By comparing the results of each model, an optimal model for endometrial cancer screening was obtained.

All of the data were analyzed by SPSS 22.0 (IBM Corp., Armonk, NY, USA) and R-program (version 3.2.2, The R Foundation for Statistical Computing).

## Results

### Synthesis and Characterization of ZW-FA

**Figure 1:**
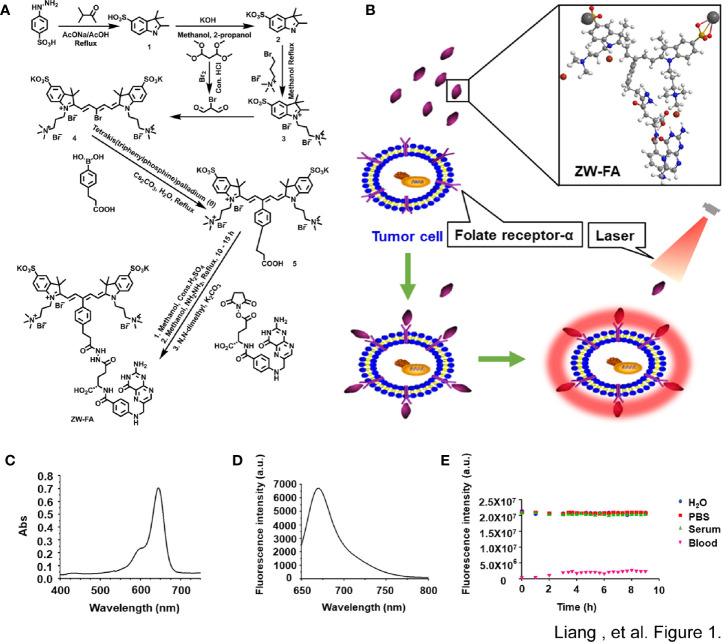
The synthesis and Characterization of ZW-FA. **(A)** Synthesis steps of ZW-FA. **(B)** Schematic diagram of the working principle for detecting endometrial cells and the ball-and-stick model of ZW-FA. ZW-FA bound to the FR-α of cell, and the ZW-FA-cell complex could emit near-infrared fluorescence with 633nm laser. **(C)** Ultraviolet spectra of ZW-FA (7.14 ug/ml). **(D)** Fluorescence spectra of ZW-FA (7.14 ug/ml) at 668 nm. λ_ex_=633 nm, slit width: d_em_=10.0 nm, d_ex_=2.5 nm. **(E)** In a variety of biological media at 37°C under continuous laser exposure at 650-850 nm.

The synthesis procedure of ZW-FA was presented in **Figure 1A** and **Supplementary Table 1**. The schematic diagram of the working principle for detecting endometrial cells and the ball-and-stick model of ZW-FA were shown in **Figure 1B**. NIR fluorophore ZW-FA was synthesized by employing 4-hydrazinobenzenesulfonic acid, potassium hydroxide and preparation of activated folic acid. The crude product was washed with eluent consisting of acetone and water with a volume ratio of 1:3. ZW-FA, the final product, had superior aqueous solubility when methanol was used as a solvent. The purity of all compounds was > 95% determined by ^1^H NMR (**Supplementary Figure 1**).

As implied in **Figures 1C, D**, ZW-FA emits near-infrared. ZW-FA had the strongest absorption peak at 633 nm in ultraviolet spectra. The fluorescence intensity of ZW-FA reached a peak at 668 nm in fluorescence spectra. We observed excellent photostability of ZW-FA by exposing it in a variety of biological media, which included H_2_O, PBS, serum, and blood (**Figure 1E**), at 37°C (8 hours).

### Fluorescence Imaging of Cell Lines

**Figure 2:**
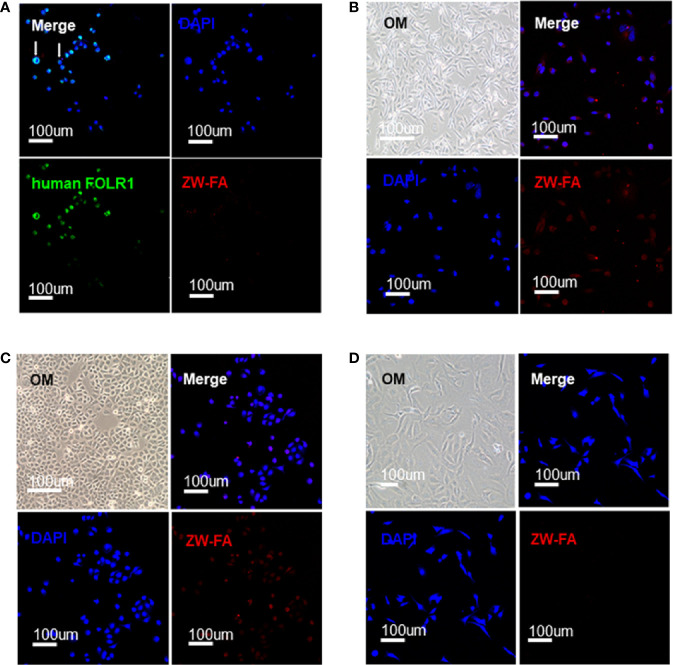
Targeting verification. **(A)** Fluorescence images of SKOV_3_, cells were incubated with human FOLR1 antibody, ZW-FA and DAPI in sequence. **(B)** Fluorescence images of MDA-MB-231, cells were incubated with ZW-FA and DAPI in sequence. **(C)** Fluorescence images of SKOV_3_, cells were incubated with ZW-FA and DAPI in sequence. **(D)** Fluorescence images of HUVEC, cells were incubated with ZW-FA and DAPI in sequence. The concentration of ZW-FA was 600 ug/ml in the three cell lines. The excitation wavelengths of DAPI, human FOLR1 antibody and ZW-FA fluorescence were 408 nm, 488 nm and 633 nm, respectively.

FR-α positive cells SKOV_3_ were incubated successively with human FOLR1 antibody, Alexa Fluor 488 and ZW-FA (600 ug/ml). The fluorescence signal in the green channel was strong, while the fluorescence signal in the red channel was weak (**Figure 2A**). The green and red channels indicated the bond of the human FOLR1 antibody and ZW-FA with FR-α of cells, respectively. The results demonstrated that the FR-α on the cell surface was firstly occupied by the human FOLR1 antibody, resulting in the binding of ZW-FA was blocked. It has been proved that ZW-FA had the ability for targeting FR-α.

FR-α positive cells SKOV_3_ and MDA-MB-231, and FR-α negative cells HUVEC were incubated with ZW-FA (600 ug/ml). The red channel of FR-α positive cells SKOV_3_ and MDA-MB-231 showed a strong fluorescence signal (**Figures 2B, C**). However, there was a weak fluorescence signal in the red channel of FR-α negative cells HUVEC (**Figure 2D**). The fluorescence signal of the red channel indicated the binding of ZW-FA with FR-α of cells. The results demonstrated that FR-α positive cell lines SKOV_3_ and MDA-MB-231 had effectively bound with ZW-FA while the FR-α negative cell line HUVEC had not.

**Figure 3:**
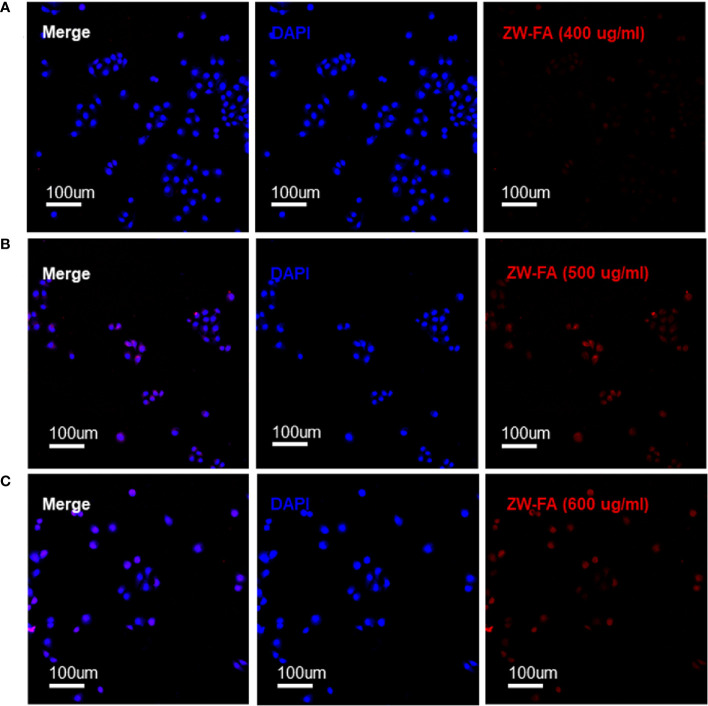
Fluorescence images of SKOV_3_ incubated with ZW-FA in gradient concentration. **(A)** Fluorescence images of SKOV_3_, cells were incubated with ZW-FA in 400 ug/ml and DAPI in sequence. **(B)** Fluorescence images of SKOV_3_, cells were incubated with ZW-FA in 500 ug/ml and DAPI in sequence. **(C)** Fluorescence images of SKOV_3_, cells were incubated with ZW-FA in 600 ug/ml and DAPI in sequence.

FR-α positive cells SKOV_3_ were incubated with ZW-FA at 400 ug/ml, 500 ug/ml and 600 ug/ml. Fluorescence intensity of the red channel increased significantly with the increase of ZW-FA concentration, which implied the concentration-dependent of ZW-FA (**Figure 3**).

### Patient Characteristics

A total of 92 patients were enrolled. The mean ± SD age was 50.97 ± 9.82, and the range was 28-74 years. According to the endometrial histopathological diagnosis system, there were 60 cases of endometrial carcinoma, 5 cases of endometrial atypical hyperplasia, 8 cases of simple endometrial hyperplasia, 11 cases of proliferative endometrium, 5 cases of atrophic endometrium, and 3 cases of secretory endometrium. Detailed information was shown in **Table 1**. The diagnosis of ZW-FA was not significantly related to age, tumor type, International Federation of Gynecology and Obstetrics (FIGO) stage, grade and depth of myometrial invasion, but was significantly related to diseases (P<0.001).

**Table 1:** Clinical characteristics of patients (N = 92).

	Total, n (%)	Diagnosis of ZW-FA (n %)		P Value
	(n = 92)	(+)	(-)	
**Age (MEAN ± SD)**	50.97 ± 9.82			0.145
<50	40 (43%)	27 (68%)	13 (32%)	
≥50	52 (57%)	42 (81%)	10 (19%)	
**Diseases**				<0.001
Noncancerous	27 (29%)	10 (37%)	17 (63%)	
Atypical	5 (6%)	5 (100%)	0 (0%)	
Cancer	60 (65%)	54 (90%)	6 (10%)	
**Tumor type**				0.238
Endometrioid	52 (87%)	47 (90%)	5 (10%)	
Serous	2 (3%)	1 (50%)	1 (50%)	
Clear cell	1 (2%)	1 (100%)	0 (0%)	
Other	5 (8%)	5 (100%)	0 (0%)	
**FIGO stage (2018)**				0.115
I	48 (80%)	44 (92%)	4 (8%)	
II	6 (10%)	6 (100%)	0 (0%)	
III	5 (8%)	3 (60%)	2 (40%)	
Unknow	1 (2%)	1 (100%)	0 (%)	
**Grade**				0.804
1	9 (15%)	8 (89%)	1 (11%)	
2	30 (50%)	28 (93%)	2 (7%)	
3	9 (15%)	8 (89%)	1 (11%)	
Unknow	12 (20%)	10 (83%)	2 (17%)	
**Depth of myometrial invasion**				0.076
<50%	32 (54%)	31 (97%)	1 (3%)	
≥50%	11 (18%)	10 (91%)	1 (9%)	
Unknow	17 (28%)	13 (76%)	4 (24%)	

### Diagnostic Utility

**Figure 4:**
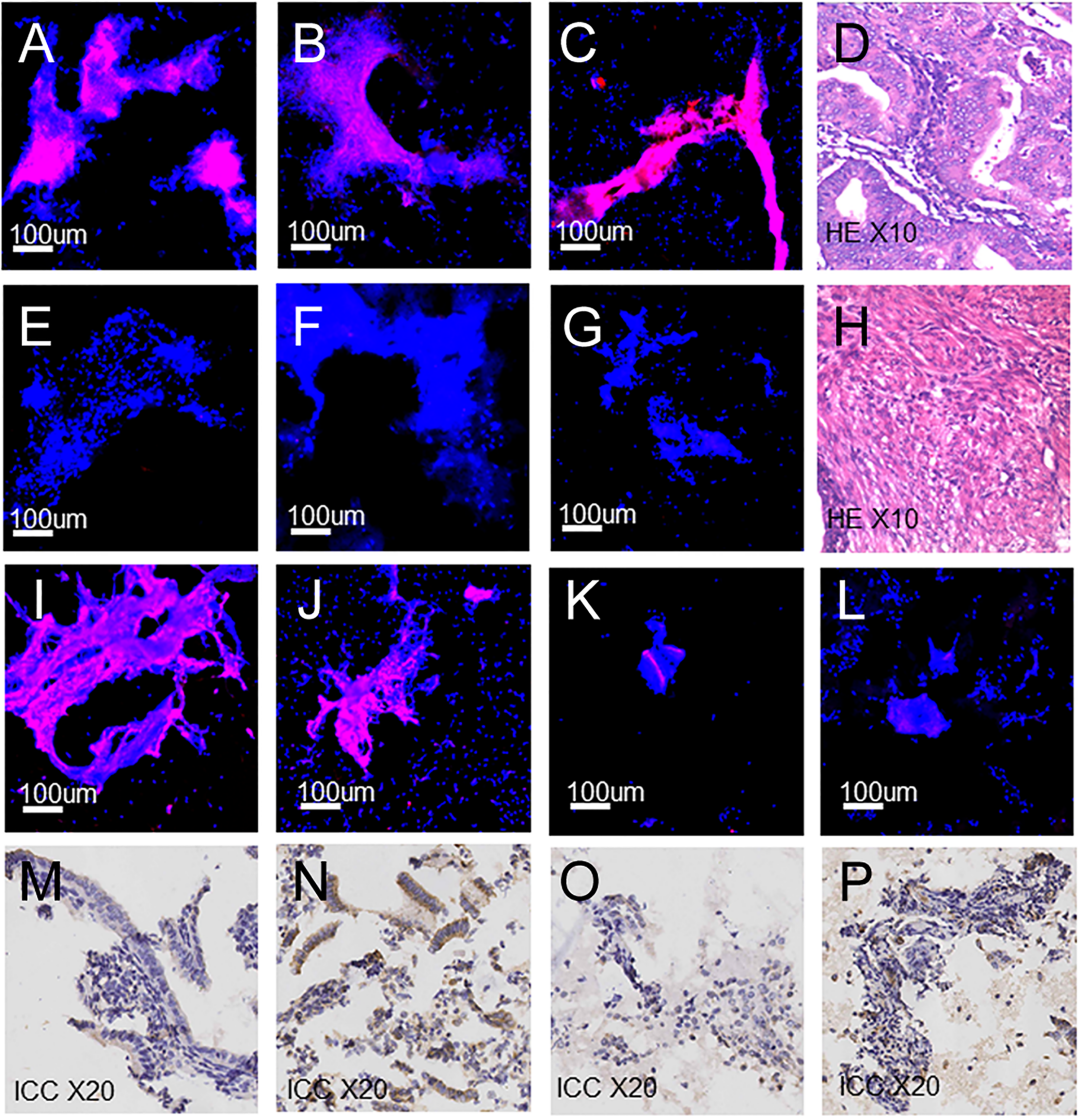
Fluorescence images, H&E and ICC images of endometrial cells. **(A–C)** Fluorescence images: **(A)** Endometrial carcinoma. **(B)** Clear-cell carcinoma. **(C)** Endometrial atypical hyperplasia. **(D)** Endometrial carcinoma (H&E). **(E–G)** Fluorescence images: **(E)** Proliferative endometrium. **(F)** Secretory endometrium. **(G)** Atrophic endometrium. **(H)** Proliferative endometrium (H&E). **(I–K)** Fluorescence images of false-positive cases. **(L)** Fluorescence images of false-negative cases. **(M–O)** ICC images of false-positive cases: **(M)** Staining intensity 1, weak. **(N)** Staining intensity 2, moderate. **(O)** Staining intensity 0, no staining. **(P)** ICC images of false-negative cases, staining intensity 1, weak.

After collecting the fluorescence images of endometrial cytology samples (**Figure 4**), the grey values of all fluorescence images were obtained by applying Image-J software which digitalized the fluorescence images in **Supplementary Table 2**. We introduced the statistical prediction models, named as ROC, ENUM, logistic regression, SVM and decision tree, to analyze the above grey values. The code was shown in **Supplementary Table 3**.

**Figure 5:**
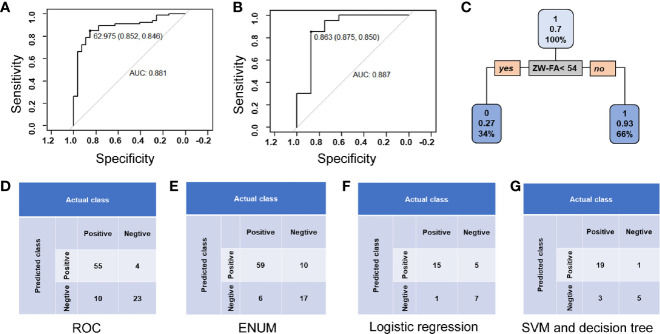
Diagnostic utility. **(A)** Curve of ROC method. **(B)** ROC curve of logistic regression. **(C)** Conditional inference tree of the training set. Patients in the training set were identified as positive, defined as 1, with a 0.93 accuracy. **(D)** Confusion matrix of the ROC method. **(E)** Confusion matrix of ENUM. **(F)** Confusion matrix of logistic regression. **(G)** Confusion matrix of SVM and decision tree.

In the ROC method, the cut-off value of ZW-FA was 62.9745, and the area under the ROC curve (AUC) was 0.881 (**Figure 5A**), which indicated the high accuracy of this test (32). According to the confusion matrix (**Figure 5D**), indicators about diagnostic utility of ZW-FA were calculated as follows: Se 84.62%, Sp 85.19%, FPR 14.81%, FNR 15.38%, PV+ 93.22% and PV– 69.70%.

In ENUM, the cut-off value of ZW-FA was 49, which was used to distinguish the type of samples. According to the confusion matrix (**Figure 5E**), indicators about diagnostic utility of ZW-FA were calculated as follows: Se 90.77%, Sp 62.96%, FPR 37.04%, FNR 9.23%, PV+ 85.51% and PV– 73.91%. Additionally, in cytopathological diagnosis, 8 out of 92 samples were considered unsatisfied sampling and 4 were missed (**Supplementary Table 2**), which implied that the new method further complemented the cytological diagnosis.

Based on the classification and recognition of machine learning, we analyzed our data by logistic regression, SVM and decision tree. The data was distinguished as a training set (64 cases) and a test set (28 cases). According to the confusion matrix (**Figure 5F**), indicators about diagnostic utility of ZW-FA from logistic regression were calculated as follows: Se 93.75%, Sp 58.33%, FPR 41.67%, FNR 6.25%, PV+ 75.00% and PV– 87.50%. AUC was 0.887, which implied a high accuracy of this test (32) (**Figure 5B**). It was shown in **Figure 5C** that the conditional inference tree of training set in decision tree model. Patients in the training set were identified as positive, defined as 1, with a 0.93 accuracy. Results of SVM and decision tree were same as follows: Se 86.36%, Sp 83.33%, FPR 16.67%, FNR 13.64%, PV+ 95.00% and PV– 62.50% (**Figure 5G**).

Generally, ROC was used to analyze data and evaluate utility in diagnostic experiments. The sensitivity and false-negative rate were calculated to be 84.62% and 15.38%, using ROC in our study initially. However, this study is dedicated to the screening of endometrial cancer and precancerous change, the high false-negative rate is not applicable. Therefore, other statistical methods were applied for building a suitable prediction model to evaluate the feasibility of ZW-FA. According to the results of models, it was believed that the ENUM was more suitable for this research. Finally, 49, cut-off value of ZW-FA from ENUM, were adopted to predict the result, and the diagnostic utility was evaluated according to the confusion matrix. We made a positive conclusion for the sample with a grey value higher than 49. Conversely, a negative conclusion was made. Thus, 69 cases were diagnosed as positive and 23 cases were negative (**Figures 4A–C, E–G**), In this study, there were 10 false-positive cases and 6 false-negative cases. The sensitivity and false-negative rate of this diagnostic test were 90.77% and 9.23%.

### Immunocytochemistry

Among the 10 false-positive cases and 6 false-negative cases, there were 5 false-positive and 2 false-negative paraffin-embedded specimens have been obtained from the sample library, and immunocytochemical staining and scoring were performed. We found that 4 out of 5 false-positive samples (**Figures 4I, J**) showed FR-α expression with staining intensity 1, weak and 2, moderate (**Figures 4M, N**). Among them, 3 cases were endometrial hyperplasia in type of postoperative pathological. There was 1 false-positive sample (**Figure 4K**) without expression of FR-α, with staining intensity 0, no staining (**Figure 4O**). FR-α expression was also shown in 2 false-negative samples (**Figure 4L**) with staining intensity 1, weak (**Figure 4P**). Detailed information is shown in **Supplementary Table 4**.

## Discussion

Screening for endometrial cancer and precancerous change is of great significance (2). In this study, we coupled folic acid and ZW800-1 to synthesis ZW-FA, which has the characteristics of targeting FR-α and emitting near-infrared. Meanwhile, we proposed a strategy for screening of endometrial cancer and precancerous change with ZW-FA. The sensitivity and false-negative rate of this strategy were 90.77% and 9.23%, and the high sensitivity and low false-negative rate are important for disease screening. Used in diagnosis *in vitro*, ZW-FA effectively identified endometrial cancer samples based on the principle of antigen-antibody specific binding.

ZW-FA has the characteristics of high hydrophilicity, emits near-infrared, and photostability. It performed excellent targeting and low non-specific binding in targeting FR-α positive and negative samples. The samples processed by ZW-FA did not affect further H&E staining and pathological diagnosis (**Supplementary Table 2**). Patients with positive results of ZW-FA would be recommended to undergo further examination for confirming the diagnosis. The strategy applied ICC and fluorescence image analysis reduced the subjective bias brought with differences in level of pathologists, and improved the objectivity of diagnosis. In addition, the method did not require pathologists to read large number of images compared with H&E, consequently reducing the work burden of cytopathologists, solving the problem of serious lack of cytopathology experts in grassroots areas, and improving screening efficiency. Thus, the current dilemma of the extreme lack of pathologists in the endometrial cytology test would be solved. Compared with immunohistochemical technology, the method was economical and convenient, and the steps were uncomplex (30). Result of diagnosis could be obtained within one day, thus the idea of rapid diagnosis was realized. To conclude, it is more suitable for rapid screening of large populations.

Expression of FR-α has increased in cancer cells of patients with endometrial cancer and other solid tumors (10–13). Scholars had experiments to distinguish between benign and malignant lesions based on the difference in FR-α expression (10, 13, 33, 34). Folic acid and its derivatives have a high affinity with FR-α, which provides a theoretical basis for complexes coupled with folic acid to target FR-α (8). FA-NIR797-MAN was made *via* combining folic acid, near-infrared fluorescent dye NIR797 and magnetic albumin nanospheres. The highly selective affinity and targeting ability to nasopharyngeal tumor cells and FR-α positive cells has been proved (35). Heptamethine cyanines, gemcitabine and folic acid were linked to synthesize a folate-receptor targeted drug, which successfully monitored the absorption of the drug through fluorescence imaging (36). Similarly, we combined folic acid with ZW800-1 to synthesize ZW-FA for targeting FR-α on the tumor cells, and proved that SKOV_3_ and MDA-MB-231 cell lines could bind effectively with high FR-α expression.

In this study, there were 10 false-positive cases, in which histopathologic diagnoses were 9 hyperplasia cases and 1 atrophic endometrium case. There were 36.1% of endometrial hyperplasia with FR-α expression, and false positives were inevitable (37). Among the 5 false-positive paraffin-embedded specimens which was treated with immunocytochemical staining and scoring, we also found that 4 out of 5 samples showed FR-α expression and 3 cases were endometrial hyperplasia in type of postoperative pathological. There were 6 false-negative cases in this study. FR-α expression was shown in 2 false-negative samples with immunocytochemical staining intensity 1. The expression level of FR-α was related to the histological type, pathological grade and FIGO stage of endometrial cancer, which indicated that not all types of endometrial cancer had significant overexpression of FR-α (38). Among the 6 false-negative cases diagnosed by cytology, 1 case was defined as an unsatisfied sample, 4 cases were of FIGO stage I and 1 case was diffuse serous carcinoma. In addition, pathologists believed that the potential reason for false negative of the unsatisfied sampling was due to little number of cells during the sampling process. We will make in-depth analysis of such cases in the future study to explore the reasons for false-negative and false-positive and improve the diagnosis process. One case were diagnosed as endometrial cancer by D&C, while postoperative histopathological examination demonstrated the result as simple hyperplasia. It is worth noting that for this case, results of ZW-FA and cytopathological were both positive. The results suggested that the method is expected to further complement the cytological diagnosis and improve the diagnosis accuracy of endometrial cancer and precancerous change.

However, a few limitations in this study are worth noting. We did not cover all histological types of the endometrium, and all the samples were from a single-center, that is the First Affiliated Hospital of Xi’an Jiaotong University. In the future, more samples and data are needed to demonstrate the feasibility of ZW-FA, and we hope to use it as an effective screening tool for endometrial cancer and precancerous change.

It was an effective cytologic strategy for *in vitro* diagnosis of endometrial cancer and precancerous change to use the targeted FR-α near-infrared fluorescent agent ZW-FA. Patients with positive diagnosis results of ZW-FA will be recommended to undergo further examination for confirming, which will greatly reduce the work burden of cytopathologists in reading pathological images, improve the screening efficiency, and make endometrial cancer and precancerous change screening hopeful.

## Data Availability Statement

The raw data supporting the conclusions of this article will be made available by the authors, without undue reservation.

## Ethics Statement

The studies involving human participants were reviewed and approved by the Ethics Committee of the First Affiliated Hospital of Xi’an Jiaotong University (XJTU1AF2017LSK-100). The patients/participants provided their written informed consent to participate in this study.

## Author Contributions

QL designed and configured the study. DL conducted the experiments and wrote the paper. XT conducted the experiments and wrote the paper. QW helped to conduct the experiments. LZ helped to conduct the experiments and polish the manuscript. KZ collected the samples. YW collected the samples. XF collected the samples. PY collected the samples. LG collected the samples. YL helped to conduct the experiments. LW collected the samples. LH collected the samples. RA helped to polish the manuscript. All authors contributed to the article and approved the submitted version.

## Funding

This work was supported by the Major Basic Research Project of Natural Science of Shaanxi Provincial Science and Technology Department (2018JM7073, 2017ZDJC-11), the Clinical Research Award of the First Affiliated Hospital of Xi’an Jiaotong University, China (XJTU1AF-2018-017, XJTU1AF-CRF-2019-002), the Key Research and Development Project of Shaanxi Provincial Science and Technology Department (2017ZDXM-SF-068, 2019QYPY-138), the Shaanxi Provincial Collaborative Technology Innovation Project (2017XT-026, 2018XT-002), and the Medical Research Project of Xi’an Social Development Guidance Plan (2017117SF/YX011-3). The funders had no role in study design, data collection and analysis, decision to publish, or preparation of the manuscript.

## Conflict of Interest

The authors declare that the research was conducted in the absence of any commercial or financial relationships that could be construed as a potential conflict of interest.

## References

[1] Koh WJ Abu-Rustum NR Bean S Bradley K Campos SM Cho KR. Uterine Neoplasms, Version 1.2018, Nccn Clinical Practice Guidelines in Oncology. J Natl Compr Canc Netw (2018) 16:170–99. 10.6004/jnccn.2018.0006 29439178

[2] Costas L Frias-Gomez J Guardiola M Benavente Y Pineda M Pavon MA. New Perspectives on Screening and Early Detection of Endometrial Cancer. Int J Cancer (2019) 145:3194–206. 10.1002/ijc.32514 31199503

[3] Fujiwara H Takahashi Y Takano M Miyamoto M Nakamura K Kaneta Y. Evaluation of Endometrial Cytology: Cytohistological Correlations in 1,441 Cancer Patients. Oncology (2015) 88:86–94. 10.1159/000368162 25324024

[4] Lee S Lim W Ryu HW Jo D Min JJ Kim HS. Zw800-1 for Assessment of Blood-Brain Barrier Disruption in a Photothrombotic Stroke Model. Int J Med Sci (2017) 14:1430–5. 10.7150/ijms.22294 PMC5707760 29200957

[5] Choi HS Nasr K Alyabyev S Feith D Lee JH Kim SH. Synthesis and *in Vivo* Fate of Zwitterionic Near-Infrared Fluorophores. Angew Chem Int Ed Engl (2011) 50:6258–63. 10.1002/anie.201102459 PMC3128676 21656624

[6] Kim KS Hyun H Yang JA Lee MY Kim H Yun SH. Bioimaging of Hyaluronate-Interferon Alpha Conjugates Using a Non-Interfering Zwitterionic Fluorophore. Biomacromolecules (2015) 16:3054–61. 10.1021/acs.biomac.5b00933 PMC4603648 26258264

[7] Verbeek FP van der Vorst JR Tummers QR Boonstra MC de Rooij KE Lowik CW. Near-Infrared Fluorescence Imaging of Both Colorectal Cancer and Ureters Using a Low-Dose Integrin Targeted Probe. Ann Surg Oncol (2014) 21(Suppl 4):S528–37. 10.1245/s10434-014-3524-x PMC4128907 24515567

[8] Scaranti M Cojocaru E Banerjee S Banerji U. Exploiting the Folate Receptor Alpha in Oncology. Nat Rev Clin Oncol (2020) 17:349–59. 10.1038/s41571-020-0339-5 32152484

[9] Elnakat H Ratnam M. Distribution, Functionality and Gene Regulation of Folate Receptor Isoforms: Implications in Targeted Therapy. Adv Drug Delivery Rev (2004) 56:1067–84. 10.1016/j.addr.2004.01.001 15094207

[10] Predina JD Newton AD Connolly C Dunbar A Baldassari M Deshpande C. Identification of a Folate Receptor-Targeted Near-Infrared Molecular Contrast Agent to Localize Pulmonary Adenocarcinomas. Mol Ther (2018) 26:390–403. 10.1016/j.ymthe.2017.10.016 29241970 PMC5835020

[11] Parker N Turk MJ Westrick E Lewis JD Low PS Leamon CP. Folate Receptor Expression in Carcinomas and Normal Tissues Determined by a Quantitative Radioligand Binding Assay. Anal Biochem (2005) 338:284–93. 10.1016/j.ab.2004.12.026 15745749

[12] Heo GS Detering L Luehmann HP Primeau T Lee YS Laforest R. Folate Receptor Alpha-Targeted (89)Zr-M9346a Immuno-Pet for Image-Guided Intervention With Mirvetuximab Soravtansine in Triple-Negative Breast Cancer. Mol Pharm (2019) 16:3996–4006. 10.1021/acs.molpharmaceut.9b00653 31369274 PMC11617356

[13] O'Shannessy DJ Somers EB Smale R Fu YS. Expression of Folate Receptor-Alpha (Fra) in Gynecologic Malignancies and Its Relationship to the Tumor Type. Int J Gynecol Pathol (2013) 32:258–68. 10.1097/PGP.0b013e3182774562 23518909

[14] Lee EK Liu JF. Antibody-Drug Conjugates in Gynecologic Malignancies. Gynecol Oncol (2019) 153:694–702. 10.1016/j.ygyno.2019.03.245 30929824

[15] Payne BK Brown-Iannuzzi JL Hannay JW. Economic Inequality Increases Risk Taking. Proc Natl Acad Sci USA (2017) 114:4643–8. 10.1073/pnas.1616453114 PMC5422783 28416655

[16] Senol S Ceyran AB Aydin A Zemheri E Ozkanli S Kosemetin D. Folate Receptor Alpha Expression and Significance in Endometrioid Endometrium Carcinoma and Endometrial Hyperplasia. Int J Clin Exp Pathol (2015) 8:5633–41. PMC4503146 26191275

[17] Altwerger G Bonazzoli E Bellone S Egawa-Takata T Menderes G Pettinella F. *In Vitro* and *in Vivo* Activity of Imgn853, an Antibody-Drug Conjugate Targeting Folate Receptor Alpha Linked to Dm4, in Biologically Aggressive Endometrial Cancers. Mol Cancer Ther (2018) 17:1003–11. 10.1158/1535-7163.MCT-17-0930 PMC5932245 29440294

[18] Xue Y Cong W Xie S Shu J Feng G Gao H. Folate-Receptor-Positive Circulating Tumor Cells as an Efficacious Biomarker for the Diagnosis of Small Pulmonary Nodules. J Cancer Res Ther (2018) 14:1620–6. 10.4103/jcrt.JCRT_905_17 30589049

[19] Martin LP Konner JA Moore KN Seward SM Matulonis UA Perez RP. Characterization of Folate Receptor Alpha (Fralpha) Expression in Archival Tumor and Biopsy Samples From Relapsed Epithelial Ovarian Cancer Patients: A Phase I Expansion Study of the Fralpha-Targeting Antibody-Drug Conjugate Mirvetuximab Soravtansine. Gynecol Oncol (2017) 147:402–7. 10.1016/j.ygyno.2017.08.015 PMC6893864 28843653

[20] Allard JE Risinger J Morrison C Young G Rose GS Fowler J. Overexpression of Folate Binding Protein Is Associated With Shortened Progression-Free Survival in Uterine Adenocarcinomas. Gynecol Oncol (2007) 107:52–7. 10.1016/j.ygyno.2007.05.018 17582475

[21] Moore KN Borghaei H O'Malley DM Jeong W Seward SM Bauer TM. Phase 1 Dose-Escalation Study of Mirvetuximab Soravtansine (Imgn853), a Folate Receptor Alpha-Targeting Antibody-Drug Conjugate, in Patients With Solid Tumors. Cancer (2017) 123:3080–7. 10.1002/cncr.30736 PMC6896318 28440955

[22] Boogerd LSF Hoogstins CES Gaarenstroom KN de Kroon CD Beltman JJ Bosse T. Folate Receptor-Alpha Targeted Near-Infrared Fluorescence Imaging in High-Risk Endometrial Cancer Patients: A Tissue Microarray and Clinical Feasibility Study. Oncotarget (2018) 9:791–801. 10.18632/oncotarget.23155 29416655 PMC5787511

[23] Corbin IR Ng KK Ding L Jurisicova A Zheng G. Near-Infrared Fluorescent Imaging of Metastatic Ovarian Cancer Using Folate Receptor-Targeted High-Density Lipoprotein Nanocarriers. Nanomed (Lond) (2013) 8:875–90. 10.2217/nnm.12.137 PMC3652907 23067398

[24] Zheng M Zhao P Luo Z Gong P Zheng C Zhang P. Robust Icg Theranostic Nanoparticles for Folate Targeted Cancer Imaging and Highly Effective Photothermal Therapy. ACS Appl Mater Interfaces (2014) 6:6709–16. 10.1021/am5004393 24697646

[25] Tuo X Zhao L Wang Q Han L Wang Y Ma S. Validation of Molecular Typing for Endometrial Screening Test That Predicts Benign and Malignant Lesions. Front Oncol (2019) 9:561. 10.3389/fonc.2019.00561 31338322 PMC6629861

[26] Lv S Wang R Wang Q Han L Tuo X Hou H. Li Q. A Novel Solution Configuration on Liquid-Based Endometrial Cytology. PloS One (2018) 13:e0190851. 10.1371/journal.pone.0190851 29401497 PMC5798778

[27] Han L Du J Zhao L Sun C Wang Q Tuo X. An Efficacious Endometrial Sampler for Screening Endometrial Cancer. Front Oncol (2019) 9:67. 10.3389/fonc.2019.00067 30838173 PMC6389657

[28] Wang Q Wang Q Zhao L Han L Sun C Ma S. Endometrial Cytology as a Method to Improve the Accuracy of Diagnosis of Endometrial Cancer: Case Report and Meta-Analysis. Front Oncol (2019) 9:256. 10.3389/fonc.2019.00256 31069167 PMC6491702

[29] Kurman RJ Kaminski PF Norris HJ. The Behavior of Endometrial Hyperplasia. A Long-Term Study of "Untreated" Hyperplasia in 170 Patients. Cancer (1985) 56:403–12. 10.1002/1097-0142(19850715)56:2<403::AID-CNCR2820560233>3.0.CO;2-X 4005805

[30] Boogerd LS Boonstra MC Beck AJ Charehbili A Hoogstins CE Prevoo HA. Concordance of Folate Receptor-Alpha Expression Between Biopsy, Primary Tumor and Metastasis in Breast Cancer and Lung Cancer Patients. Oncotarget (2016) 7:17442–54. 10.18632/oncotarget.7856 PMC4951224 26943581

[31] Henry CE Llamosas E Daniels B Coopes A Tang K Ford CE. Ror1 and Ror2 Play Distinct and Opposing Roles in Endometrial Cancer. Gynecol Oncol (2018) 148:576–84. 10.1016/j.ygyno.2018.01.025 29395309

[32] Xiong F Xiao J Bai Y Zhang Y Li Q Lishuang X. Metformin Inhibits Estradiol and Progesterone-Induced Decidualization of Endometrial Stromal Cells by Regulating Expression of Progesterone Receptor, Cytokines and Matrix Metalloproteinases. BioMed Pharmacother (2019) 109:1578–85. 10.1016/j.biopha.2018.10.128 30551411

[33] Wu M Gunning W Ratnam M. Expression of Folate Receptor Type Alpha in Relation to Cell Type, Malignancy, and Differentiation in Ovary, Uterus, and Cervix. Cancer Epidemiol Biomarkers Prev (1999) 8:775–82. 10498396

[34] Dainty LA Risinger J Morrison C Chandramouli GV Bidus M Zahn C. Overexpression of Folate Binding Protein and Mesothelin Are Associated With Uterine Serous Carcinoma. Gynecol Oncol (2007) 105:563–70. 10.1016/j.ygyno.2006.10.063 17400285

[35] Tang Q An Y Liu D Liu P Zhang D. Folate/Nir 797-Conjugated Albumin Magnetic Nanospheres: Synthesis, Characterisation, and *in Vitro* and *in Vivo* Targeting Evaluation. PloS One (2014) 9:e106483. 10.1371/journal.pone.0106483 25188308 PMC4154716

[36] Yang Z Lee JH Jeon HM Han JH Park N He Y. Folate-Based Near-Infrared Fluorescent Theranostic Gemcitabine Delivery. J Am Chem Soc (2013) 135:11657–62. 10.1021/ja405372k 23865715

[37] Wang J Jia N Lyv T Wang C Tao X Wong K. Paired Box 2 Promotes Progression of Endometrial Cancer Via Regulating Cell Cycle Pathway. J Cancer (2018) 9:3743–54. 10.7150/jca.22418 PMC6216001 30405846

[38] Li Q Zhang J Liang Y Mu W Hou X Ma X. Tanshinone L Exhibits Anticancer Effects in Human Endometrial Carcinoma Hec-1-a Cells Via Mitochondrial Mediated Apoptosis, Cell Cycle Arrest and Inhibition of Jak/Stat Signalling Pathway. J BUON (2018) 23:1092–6. 30358216

