# Supplementary material for: Near-Infrared Fluorescent Agent for In Vitro Screening of Endometrial Cancer and Precancerous Lesions
Source: Front Oncol. 2021 Jul 1;11:713583. doi: 10.3389/fonc.2021.713583 (PMC8281888; doi:10.3389/fonc.2021.713583)

***Supplementary Material***

**Supplementary Table 1. Synthesis of ZW-FA.**

**2,3,3-Trimethylindolenine-5-sulfonic acid (1)**

A stirred solution of p-hydrazinobenzenesulfonic acid (5.0 g, 26.5 mmol) in acetic acid (15 ml) was treated with 3-methyl-2-butanone (8.3 ml, 78 mmol) at 25 °C. The reaction mixture was heated to reflux for 3 h, then cooled down to 25 °C and filtered through a paper filter. The residue was washed with ethyl acetate and dried under reduced pressure to provide the sulfonic acid as a pink powder **1** (5.2 g, 82%), which was used in the next step without further purification. ^1^H NMR (400 MHz, D_2_O, ppm): *δ* 7.86 (s, 1H), 7.79 (d, *J*=8.0 Hz 1H), 7.57 (d, *J*=8.4 Hz, 1H), 1.36 (s, 9H). ESI-MS: *m*/*z*, 240.0691, [M+H]^+^; 238.0572, [M-H] −.

**2,3,3-Trimethylindolenine-5-sulfonic acid, potassium salt (2)**

A solution of crude sulfonic acid **1** (5.0 g, 20 mmol) in methanol (6 ml) was added dropwise to a stirred solution of potassium hydroxide (1.76 g, 31.4 mmol) in isopropanol (35 ml). The resulting mixture was stirred at 25 °C for 24 h and filtered through a paper filter. The residue was dried under reduced pressure to provide a yellow powder **2** (4.9 g, 88%). ^1^H NMR (400 MHz, DMSO-*d_6_*, ppm): *δ* 7.61 (s, 1H), 7.54 (d, *J*=8.4 Hz 1H), 7.34 (d, *J*=7.2 Hz, 1H), 2.20 (s, 3H), 1.24 (s, 6H).

**2,3,3-Trimethyl-1-[3-(trimethylammonio)propyl]-3H-indolium-5-sulfonic acid dibromide (3)**

A mixture of 2,3,3-trimethyl-3*H*-indole-5-sulfonic acid **2** (2.66 g, 9.6 mmol) and (3-bromopropyl) trimethyl ammonium bromide (2.75 g, 10.5 mmol) in methanol (22 ml) was heated at 130 °C for 72 h under a nitrogen atmosphere. The mixture was cooled to room temperature and the solvent was decanted. The crude product was crystallized from methanol and MTBE to afford pink crystals **3**, which was used in the next step without further purification (3.0 g, 58%); ^1^H NMR (400 MHz, DMSO-*d_6_*, ppm): *δ* 8.04 (s, 1H), 7.99 (d, *J*=8.0 Hz 1H), 7.78 (d, *J*=8.0 Hz, 1H), 4.48 (s, 2H), 3.09 (s, 9H), 3.03 (s, 2H), 2.89 (s, 3H), 2.32 (s, 2H), 1.56 (s, 6H). C_17_H_27_N_2_O_3_S [M]^+^ *m/z* 33 9.1740, found *m/z* 339.1736.

**Bromomalonaldehyde**

1,1,3,3-Tetramethoxypropane (10.66 g., 55 mmol) was stirred with 13 ml of water and 0.43 ml of concentrated hydrochloric acid until a homogeneous solution resulted. At 0 °C, 30 ml CCl_4_ solution of Br_2_ (3.38 ml) was added slowly to the stirred solution. After the addition was complete, the clear yellow solution was concentrated at reduced pressure to yield thick slurry. Then it was filtered through a paper filter to provide bromomalonaldehyde as yellowish powder. The powder was washed with cold 50% ethanol and dried reduced pressure. The yield was 4 g (48%). Mp: 143-145. ^1^H NMR (400 MHz, DMSO-*d_6_*,, ppm): *δ* 8.71 (s, 1H).

**Compound 4**

To a solution of compound **3** (1.20 g, 5.0 mmol) was added bromomalonaldehyde (0.23 g, 1.5 mmol) in 5 ml of MeOH. This solution was sealed in a thick-walled glass pressure reactor and heated to 60-70 °C for 6 h. After cooling, the dark blue solution was filtered to provide a blue powder **4** (1.25 g, 75%), which was used in the next step without further purification. ^1^H NMR (400 MHz, D_2_O): δ 8.15 (d, J = 13.2 Hz, 1H), 7.88 (s, 1H), 7.82 (d, J = 8.8 Hz, 1H), 7.37 (d, J= 7.6 Hz, 1H), 6.40 (d, J = 13.6 Hz, 1H), 4.29 (s, 2H), 3.51 (t, 2H), 3.11 (s, 9H), 2.35 (s, 2H), 1.67 (s, 6H).

**Compound 5**

Compound **4** (0.22 g, 0.2 mmol), 4-(2-carboxyethyl)phenylboronic acid (50 mg, 0.26 mmol), and cesium carbonate (40 mg) were stirred in water (40 ml) under nitrogen at room temperature. Tetrakis(triphenylphospine)palladium(0) (23 mg, 0.02 mmol) were added to the reaction mixture. The mixture was refluxed for 4h, then the solvent and volatile compounds were evaporated under vacuum. The crude product was purified by flash chromatography on silica, eluting with a 50/50 (V/V) acetonitrile/water mixture, yield compound **5** (0.1 g, 42.3%). ^1^H NMR (400 MHz, D_2_O): δ 8.20 (d, J = 13.2 Hz, 2H), 7.75 (s, 2H), 7.68 (d, J = 5.6 Hz, 2H), 7.48(s, 2H), 7.17 (s, 2H), 6.93 (d, J= 4.4 Hz, 2H), 5.56 (d, J = 12.8 Hz, 2H), 3.59 (s, 4H), 3.13 (t, 4H), 2.96 (s, 18H), 2.58 (s, 2H), 1.90 (s, 4H), 1.58 (s, 12H). ESI-MS:C_46_H_61_N_4_O_8_S^2+^ [M]^+^ m/z 861.3925, found m/z 861.3956.C_46_H_60_N_4_O_8_S_2_ [M-H]^-^ m/z 859.3779, found m/z 859.3715.

**Preparation of activated folic acid**

Under the dark environment, folate (3 g, 6.8 mmol) was reacted with N,N′-dicyclohexylcarbodiimide (DCC) (1 g, 4.9 mmol) in DMF (60 ml). After stirring at room temperature, nhydroxysuccinimide (NHS) (0.86 g, 7.5 mmol) was added to the mixture, keep stirring at room temperature for 1 day. The product mixture was then filtered to remove residue, then added 300 ml acetone/diethyl ether (3/7, V/V) to the filter liquor. And the precipitate thus formed was filtered off, to give a yellow solid **6** (3.11 g, 5.8 mmol, 85%).

**Folic Acid-ZW800-1 (ZW-FA)**

25 ml concentrated H_2_SO_4_ (14 mol/L) was added dropwise to 500 ml methanol solution which containing compound **5** (5 g, 4.2 mmol ) at 0 °C . Then the solution was stirring and reflux for 5 h. Then use sodium bicarbonate to adjust pH of the system to 6, and add 500 ml methyl alcohol and 15 ml hydrazine hydrate after removing the solvent, the mixture refluxing for 15 h and then remove the solvent by rotary evaporation. Add 150 ml N,N-Dimethylformamide, 1 g potassium carbonate and 3 g folic acid activated by NHS to the residue after rotary evaporation. The mixture reacts for 3 days at room temperature, remove the solvent again by rotary evaporation after that, and purify the crude product by column chromatography. NMR and MS-ESI are used to detect the target product during column chromatography and the eluent is consisted of acetone and water with a volume ratio of 1:3. The near infrared fluorescence dye with the structural formula 1 is obtained with a yield 82.5%. ^1^H NMR (400 MHz, D_2_O): δ 8.45 (s, 1H), 7.68 (m, 1H), 7.57 (d, J = 8.7 Hz, 2H), 7.52(d, J = 9.1 Hz, 1H), 7.40(s, 1H) 7.47(d, J = 9.1 Hz, 1H), 7.37 (s, 1H), 7.24 (d, J= 7.6 Hz, 2H), 6.99(d, J = 7.6 Hz, 2H) 6.87(t, J = 15.2 Hz, 1H) 6.69(d, J = 8.7 Hz, 2H) 6.54 (d, J = 8.7 Hz, 1H) 6.39(d, J = 8.2 Hz, 1H) 5.04 (d, J = 15.6 Hz, 1H), 4.91(d, J = 15.6 Hz, 1H) 4.44 (s, 2H), 4.21 (m, 1H), 3.20-2.80 (m, 8H), 2.88 (s, 9H) 2.85(s, 9H), 2.43(t, J = 7.7 Hz, 2H) 2.22(t, J = 7.7 Hz, 2H) 2.20-2.00 (m, 2H), 2.00-1.85 (m, 2H), 1.85-1.50 (m, 4H), 1.42(s, 3H), 1.36 (s, 3H), 1.27(s, 3H), 1.22(s, 3H). ^13^C NMR (400 MHz, H_2_O): 181.9, 181.4, 181.1, 178.8, 177.4, 172.6, 169.2, 163.5, 155.0, 150.5, 149.4, 146.8, 146.7, 128.6, 128.0, 127.7, 121.2, 119.7, 112.1, 108.5, 63.3, 55.4, 53.2, 45.4, 38.5, 36.4, 33.8, 31.1, 28.0, 26.9, 26.8, 25.3, 22.4, 20.5, 18.1. IR(cm^−1^): 3564m, 3411m, 3320m, 3114m, 2929m, 1691s, 1606s, 1482s, 1413m, 1186m, 1106m, 1020m, 919m. ESI-MS:C_65_H_80_N_13_O_12_S_2_^2+^ [M]^+^ m/z 1299.5564, found m/z 1299.5586.

**Supplementary Table 2. Cytological and histological diagnosis patients.**

| Number | Histopathologic diagnosis | Histopathologic group | ZW-FA fluorescence intensity | ZW-FA group | Cytopathologic diagnosis | Cytopathologic group |
| --- | --- | --- | --- | --- | --- | --- |
| 1 | endometrial carcinoma | 1 | 83.294 | 1 | unsatisfied sampling | / |
| 2 | endometrial carcinoma | 1 | 82.32 | 1 | unsatisfied sampling | / |
| 3 | endometrial carcinoma | 1 | 80.94 | 1 | unsatisfied sampling | / |
| 4 | endometrial carcinoma | 1 | 77.577 | 1 | unsatisfied sampling | / |
| 5 | endometrial carcinoma | 1 | 8.105 | 0 | unsatisfied sampling | / |
| 6 | endometrial carcinoma | 1 | 73.085 | 1 | endometrial hyperplasia cells | 0 |
| 7 | endometrial carcinoma | 1 | 59.107 | 1 | endometrial hyperplasia cells | 0 |
| 8 | endometrial carcinoma | 1 | 81.386 | 1 | endometrial atypical cells | 1 |
| 9 | endometrial carcinoma | 1 | 77.011 | 1 | endometrial atypical cells | 1 |
| 10 | endometrial carcinoma | 1 | 74.607 | 1 | endometrial atypical cells | 1 |
| 11 | endometrial carcinoma | 1 | 83.44 | 1 | endometrial cancer cells | 1 |
| 12 | endometrial carcinoma | 1 | 82.609 | 1 | endometrial cancer cells | 1 |
| 13 | endometrial carcinoma | 1 | 82.569 | 1 | endometrial cancer cells | 1 |
| 14 | endometrial carcinoma | 1 | 82.435 | 1 | endometrial cancer cells | 1 |
| 15 | endometrial carcinoma | 1 | 82.33 | 1 | endometrial cancer cells | 1 |
| 16 | endometrial carcinoma | 1 | 82.055 | 1 | endometrial cancer cells | 1 |
| 17 | endometrial carcinoma | 1 | 81.882 | 1 | endometrial cancer cells | 1 |
| 18 | endometrial carcinoma | 1 | 81.7 | 1 | endometrial cancer cells | 1 |
| 19 | endometrial carcinoma | 1 | 81.682 | 1 | endometrial cancer cells | 1 |
| 20 | endometrial carcinoma | 1 | 81.182 | 1 | endometrial cancer cells | 1 |
| 21 | endometrial carcinoma | 1 | 80.433 | 1 | endometrial cancer cells | 1 |
| 22 | endometrial carcinoma | 1 | 79.939 | 1 | endometrial cancer cells | 1 |
| 23 | endometrial carcinoma | 1 | 79.447 | 1 | endometrial cancer cells | 1 |
| 24 | endometrial carcinoma | 1 | 78.81 | 1 | endometrial cancer cells | 1 |
| 25 | endometrial carcinoma | 1 | 78.721 | 1 | endometrial cancer cells | 1 |
| 26 | endometrial carcinoma | 1 | 78.367 | 1 | endometrial cancer cells | 1 |
| 27 | endometrial carcinoma | 1 | 77.913 | 1 | endometrial cancer cells | 1 |
| 28 | endometrial carcinoma | 1 | 77.753 | 1 | endometrial cancer cells | 1 |
| 29 | endometrial carcinoma | 1 | 76.896 | 1 | endometrial cancer cells | 1 |
| 30 | endometrial carcinoma | 1 | 76.735 | 1 | endometrial cancer cells | 1 |
| 31 | endometrial carcinoma | 1 | 75.399 | 1 | endometrial cancer cells | 1 |
| 32 | endometrial carcinoma | 1 | 75.263 | 1 | endometrial cancer cells | 1 |
| 33 | endometrial carcinoma | 1 | 75.025 | 1 | endometrial cancer cells | 1 |
| 34 | endometrial carcinoma | 1 | 74.664 | 1 | endometrial cancer cells | 1 |
| 35 | endometrial carcinoma | 1 | 73.085 | 1 | endometrial cancer cells | 1 |
| 36 | endometrial carcinoma | 1 | 72.482 | 1 | endometrial cancer cells | 1 |
| 37 | endometrial carcinoma | 1 | 72.183 | 1 | endometrial cancer cells | 1 |
| 38 | endometrial carcinoma | 1 | 71.607 | 1 | endometrial cancer cells | 1 |
| 39 | endometrial carcinoma | 1 | 71.127 | 1 | endometrial cancer cells | 1 |
| 40 | endometrial carcinoma | 1 | 69.978 | 1 | endometrial cancer cells | 1 |
| 41 | endometrial carcinoma | 1 | 69.681 | 1 | endometrial cancer cells | 1 |
| 42 | endometrial carcinoma | 1 | 68.989 | 1 | endometrial cancer cells | 1 |
| 43 | endometrial carcinoma | 1 | 68.981 | 1 | endometrial cancer cells | 1 |
| 44 | endometrial carcinoma | 1 | 67.974 | 1 | endometrial cancer cells | 1 |
| 45 | endometrial carcinoma | 1 | 67.796 | 1 | endometrial cancer cells | 1 |
| 46 | endometrial carcinoma | 1 | 66.079 | 1 | endometrial cancer cells | 1 |
| 47 | endometrial carcinoma | 1 | 64.67 | 1 | endometrial cancer cells | 1 |
| 48 | endometrial carcinoma | 1 | 63.571 | 1 | endometrial cancer cells | 1 |
| 49 | endometrial carcinoma | 1 | 63.487 | 1 | endometrial cancer cells | 1 |
| 50 | endometrial carcinoma | 1 | 55.747 | 1 | endometrial cancer cells | 1 |
| 51 | endometrial carcinoma | 1 | 54.847 | 1 | endometrial cancer cells | 1 |
| 52 | endometrial carcinoma | 1 | 49.645 | 1 | endometrial cancer cells | 1 |
| 53 | endometrial carcinoma | 1 | 39.919 | 0 | endometrial cancer cells | 1 |
| 54 | endometrial carcinoma | 1 | 29.496 | 0 | endometrial cancer cells | 1 |
| 55 | endometrial carcinoma | 1 | 25.321 | 0 | endometrial cancer cells | 1 |
| 56 | endometrial carcinoma | 1 | 23.928 | 0 | endometrial cancer cells | 1 |
| 57 | endometrial carcinoma | 1 | 23.278 | 0 | endometrial cancer cells | 1 |
| 58 | endometrial carcinoma | 1 | 87.273 | 1 | atrophic endometrial cells | 0 |
| 59 | endometrial carcinoma | 1 | 64.259 | 1 | atrophic endometrial cells | 0 |
| 60 | simple hyperplasia** | 1 | 75.988 | 1 | endometrial cancer cells | 1 |
| 61 | simple hyperplasia | 0 | 80.424 | 1 | unsatisfied sampling | / |
| 62 | simple hyperplasia | 0 | 4 | 0 | unsatisfied sampling | / |
| 63 | simple hyperplasia | 0 | 44.153 | 0 | proliferative endometrial cells | 0 |
| 64 | simple hyperplasia | 0 | 7.947 | 0 | proliferative endometrial cells | 0 |
| 65 | simple hyperplasia | 0 | 65.534 | 1 | endometrial hyperplasia cells | 0 |
| 66 | simple hyperplasia | 0 | 50.411 | 1 | endometrial hyperplasia cells | 0 |
| 67 | simple hyperplasia | 0 | 6.839 | 0 | endometrial hyperplasia cells | 0 |
| 68 | simple hyperplasia | 0 | 4.255 | 0 | endometrial hyperplasia cells | 0 |
| 69 | endometrial atypical hyperplasia | 1 | 66.743 | 1 | endometrial atypical cells | 1 |
| 70 | endometrial atypical hyperplasia | 1 | 80.031 | 1 | endometrial atypical cells | 1 |
| 71 | endometrial atypical hyperplasia | 1 | 71.461 | 1 | endometrial atypical cells | 1 |
| 72 | endometrial atypical hyperplasia | 1 | 79.579 | 1 | endometrial cancer cells | 1 |
| 73 | endometrial atypical hyperplasia | 1 | 82.289 | 1 | endometrial cancer cells | 1 |
| 74 | atrophic endometrium | 0 | 53.214 | 1 | atrophic endometrial cells | 0 |
| 75 | atrophic endometrium | 0 | 46.163 | 0 | atrophic endometrial cells | 0 |
| 76 | atrophic endometrium | 0 | 36.639 | 0 | atrophic endometrial cells | 0 |
| 77 | atrophic endometrium | 0 | 30.434 | 0 | atrophic endometrial cells | 0 |
| 78 | atrophic endometrium | 0 | 14.615 | 0 | atrophic endometrial cells | 0 |
| 79 | proliferative endometrium | 0 | 48.148 | 0 | unsatisfied sampling | / |
| 80 | proliferative endometrium | 0 | 70.626 | 1 | proliferative endometrial cells | 0 |
| 81 | proliferative endometrium | 0 | 68.573 | 1 | proliferative endometrial cells | 0 |
| 82 | proliferative endometrium | 0 | 62.462 | 1 | proliferative endometrial cells | 0 |
| 83 | proliferative endometrium | 0 | 60.453 | 1 | proliferative endometrial cells | 0 |
| 84 | proliferative endometrium | 0 | 53.1 | 1 | proliferative endometrial cells | 0 |
| 85 | proliferative endometrium | 0 | 50.774 | 1 | proliferative endometrial cells | 0 |
| 86 | proliferative endometrium | 0 | 40.397 | 0 | proliferative endometrial cells | 0 |
| 87 | proliferative endometrium | 0 | 32.611 | 0 | proliferative endometrial cells | 0 |
| 88 | proliferative endometrium | 0 | 20.475 | 0 | proliferative endometrial cells | 0 |
| 89 | proliferative endometrium | 0 | 29.452 | 0 | endometrial hyperplasia cells | 0 |
| 90 | secretory endometrium | 0 | 46.54 | 0 | secretory endometrial cells | 0 |
| 91 | secretory endometrium | 0 | 44.309 | 0 | secretory endometrial cells | 0 |
| 92 | secretory endometrium | 0 | 12.804 | 0 | proliferative endometrial cells | 0 |

Notes:

1: "1" stand for "positive"; "0" stand for "negative"; "/" stand for "none".

2: ** In this case, D&C diagnosed it as endometrial cancer, and postoperative histopathological examination diagnosed it as simple hyperplasia. While the results of ZW-FA and cytopathological were both positive.

# Supplementary Table 3. Code.

##Data read
library(readxl)
setwd("C:/Users/lixia/Desktop/ new folder")
data=read_excel("ZW-FA-20201010.xlsx",sheet=1)


##Method1: Enumeration threshold

lline=min(data$`ZW-FA`)
hline=max(data$`ZW-FA`)
lline=floor(lline)+1
hline=ceiling(hline)-1

result=data.frame()
for(i in lline : hline){
 judge=ifelse(data$`ZW-FA`>=i,1,0)
 cfm=table(judge,data$`Histopathologic diagnosis`)
 acc=sum(diag(cfm))/sum(cfm)
 ppv=cfm[2,2]/(cfm[2,1]+cfm[2,2])
 tpr=cfm[2,2]/(cfm[1,2]+cfm[2,2])
 tnr=cfm[1,1]/(cfm[1,1]+cfm[2,1])
 result[i,1]=acc
 result[i,2]=ppv
 result[i,3]=tpr
 result[i,4]=tnr
}
names(result)=c("acc","ppv","tpr","tnr")
result

## acc ppv tpr tnr
## 1 NA NA NA NA
## 2 NA NA NA NA
## 3 NA NA NA NA
## 4 NA NA NA NA
## 5 0.7282609 0.7222222 1.00000000 0.07407407
## 6 0.7282609 0.7222222 1.00000000 0.07407407
## 7 0.7391304 0.7303371 1.00000000 0.11111111
## 8 0.7500000 0.7386364 1.00000000 0.14814815
## 9 0.7391304 0.7356322 0.98461538 0.14814815
## 10 0.7391304 0.7356322 0.98461538 0.14814815
## 11 0.7391304 0.7356322 0.98461538 0.14814815
## 12 0.7391304 0.7356322 0.98461538 0.14814815
## 13 0.7500000 0.7441860 0.98461538 0.18518519
## 14 0.7500000 0.7441860 0.98461538 0.18518519
## 15 0.7608696 0.7529412 0.98461538 0.22222222
## 16 0.7608696 0.7529412 0.98461538 0.22222222
## 17 0.7608696 0.7529412 0.98461538 0.22222222
## 18 0.7608696 0.7529412 0.98461538 0.22222222
## 19 0.7608696 0.7529412 0.98461538 0.22222222
## 20 0.7608696 0.7529412 0.98461538 0.22222222
## 21 0.7717391 0.7619048 0.98461538 0.25925926
## 22 0.7717391 0.7619048 0.98461538 0.25925926
## 23 0.7717391 0.7619048 0.98461538 0.25925926
## 24 0.7500000 0.7560976 0.95384615 0.25925926
## 25 0.7500000 0.7560976 0.95384615 0.25925926
## 26 0.7391304 0.7530864 0.93846154 0.25925926
## 27 0.7391304 0.7530864 0.93846154 0.25925926
## 28 0.7391304 0.7530864 0.93846154 0.25925926
## 29 0.7391304 0.7530864 0.93846154 0.25925926
## 30 0.7391304 0.7594937 0.92307692 0.29629630
## 31 0.7500000 0.7692308 0.92307692 0.33333333
## 32 0.7500000 0.7692308 0.92307692 0.33333333
## 33 0.7608696 0.7792208 0.92307692 0.37037037
## 34 0.7608696 0.7792208 0.92307692 0.37037037
## 35 0.7608696 0.7792208 0.92307692 0.37037037
## 36 0.7608696 0.7792208 0.92307692 0.37037037
## 37 0.7717391 0.7894737 0.92307692 0.40740741
## 38 0.7717391 0.7894737 0.92307692 0.40740741
## 39 0.7717391 0.7894737 0.92307692 0.40740741
## 40 0.7608696 0.7866667 0.90769231 0.40740741
## 41 0.7717391 0.7972973 0.90769231 0.44444444
## 42 0.7717391 0.7972973 0.90769231 0.44444444
## 43 0.7717391 0.7972973 0.90769231 0.44444444
## 44 0.7717391 0.7972973 0.90769231 0.44444444
## 45 0.7934783 0.8194444 0.90769231 0.51851852
## 46 0.7934783 0.8194444 0.90769231 0.51851852
## 47 0.8152174 0.8428571 0.90769231 0.59259259
## 48 0.8152174 0.8428571 0.90769231 0.59259259
## 49 0.8260870 0.8550725 0.90769231 0.62962963
## 50 0.8152174 0.8529412 0.89230769 0.62962963
## 51 0.8369565 0.8787879 0.89230769 0.70370370
## 52 0.8369565 0.8787879 0.89230769 0.70370370
## 53 0.8369565 0.8787879 0.89230769 0.70370370
## 54 0.8586957 0.9062500 0.89230769 0.77777778
## 55 0.8478261 0.9047619 0.87692308 0.77777778
## 56 0.8369565 0.9032258 0.86153846 0.77777778
## 57 0.8369565 0.9032258 0.86153846 0.77777778
## 58 0.8369565 0.9032258 0.86153846 0.77777778
## 59 0.8369565 0.9032258 0.86153846 0.77777778
## 60 0.8260870 0.9016393 0.84615385 0.77777778
## 61 0.8369565 0.9166667 0.84615385 0.81481481
## 62 0.8369565 0.9166667 0.84615385 0.81481481
## 63 0.8478261 0.9322034 0.84615385 0.85185185
## 64 0.8260870 0.9298246 0.81538462 0.85185185
## 65 0.8043478 0.9272727 0.78461538 0.85185185
## 66 0.8152174 0.9444444 0.78461538 0.88888889
## 67 0.7934783 0.9423077 0.75384615 0.88888889
## 68 0.7717391 0.9400000 0.72307692 0.88888889
## 69 0.7608696 0.9574468 0.69230769 0.92592593
## 70 0.7391304 0.9555556 0.66153846 0.92592593
## 71 0.7500000 0.9772727 0.66153846 0.96296296
## 72 0.7173913 0.9756098 0.61538462 0.96296296
## 73 0.6956522 0.9743590 0.58461538 0.96296296
## 74 0.6739130 0.9729730 0.55384615 0.96296296
## 75 0.6521739 0.9714286 0.52307692 0.96296296
## 76 0.6086957 0.9677419 0.46153846 0.96296296
## 77 0.5869565 0.9655172 0.43076923 0.96296296
## 78 0.5434783 0.9600000 0.36923077 0.96296296
## 79 0.5108696 0.9545455 0.32307692 0.96296296
## 80 0.4782609 0.9473684 0.27692308 0.96296296
## 81 0.4565217 1.0000000 0.23076923 1.00000000
## 82 0.4021739 1.0000000 0.15384615 1.00000000
## 83 0.3260870 1.0000000 0.04615385 1.00000000
## 84 0.3043478 1.0000000 0.01538462 1.00000000
## 85 0.3043478 1.0000000 0.01538462 1.00000000
## 86 0.3043478 1.0000000 0.01538462 1.00000000
## 87 0.3043478 1.0000000 0.01538462 1.00000000

write.csv(result,"result of Method1.csv")
###Method2: ROC

library(pROC)

## Type 'citation("pROC")' for a citation.

##
## Attaching package: 'pROC'

## The following objects are masked from 'package:stats':
##
## cov, smooth, var

roc(data$`Histopathologic diagnosis`, data$`ZW-FA`, plot=TRUE, print.thres=TRUE, print.auc=TRUE)

## Setting levels: control = 0, case = 1

## Setting direction: controls < cases

##
## Call:
## roc.default(response = data$`Histopathologic diagnosis`, predictor = data$`ZW-FA`, plot = TRUE, print.thres = TRUE, print.auc = TRUE)
##
## Data: data$`ZW-FA` in 27 controls (data$`Histopathologic diagnosis` 0) < 65 cases (data$` Histopathologic diagnosis` 1).
## Area under the curve: 0.8815

###Method3: Classification and Identification
## Distinguish between training set test set
### Distinguish between training set test set
data$`Histopathologic diagnosis`=as.factor(data$`Histopathologic diagnosis`)
split = sample(nrow(data),nrow(data)*(7/10),replace=F)
data_train = data[split,]# training
data_test = data[-split,]# test

### Logistic regression
library(pROC)
model = glm(`Histopathologic diagnosis` ~ `ZW-FA`, family = "binomial",data = data_train)
summary(model)

##
## Call:
## glm(formula = `Histopathologic diagnosis` ~ `ZW-FA`, family = "binomial",
## data = data_train)
##
## Deviance Residuals:
## Min 1Q Median 3Q Max
## -1.9265 -0.5153 0.4373 0.5805 2.0712
##
## Coefficients:
## Estimate Std. Error z value Pr(>|z|)
## (Intercept) -2.51707 0.92555 -2.720 0.00654 **
## `ZW-FA` 0.06129 0.01609 3.808 0.00014 ***
## ---
## Signif. codes: 0 '***' 0.001 '**' 0.01 '*' 0.05 '.' 0.1 ' ' 1
##
## (Dispersion parameter for binomial family taken to be 1)
##
## Null deviance: 77.849 on 63 degrees of freedom
## Residual deviance: 57.011 on 62 degrees of freedom
## AIC: 61.011
##
## Number of Fisher Scoring iterations: 4

p = predict(model,data_test,type="response")
modelroc2 = roc(data_test$`Histopathologic diagnosis`,p)

## Setting levels: control = 0, case = 1
## Setting direction: controls < cases

# Calculate AUC
(auc2 = auc(modelroc2))

## Area under the curve: 0.8875

#Draw ROC
plot(modelroc2,print.thres=TRUE,print.auc=T,main="AIC selection model",xlab="specificity",ylab="sensitivity")

## Calculation accuracy
p=ifelse(p>0.876,1,0)
ljp=table(data_test$`Histopathologic diagnosis`,p)
ljp

## p
## 0 1
## 0 7 1
## 1 5 15

p1=sum(diag(ljp))/sum(ljp)
p1

## [1] 0.7857143

#### Support vector machine (SVM)

 #
library(e1071) #

###SVM

svm1=svm(`Histopathologic diagnosis` ~ `ZW-FA`,data_train) # Building svm models
summary(svm1)

##
## Call:
## svm(formula = `Histopathologic diagnosis` ~ `ZW-FA`, data = data_train)
##
##
## Parameters:
## SVM-Type: C-classification
## SVM-Kernel: radial
## cost: 1
##
## Number of Support Vectors: 30
##
## ( 15 15 )
##
##
## Number of Classes: 2
##
## Levels:
## 0 1

pre_svm1=predict(svm1,data_test)

### Accuracy
confusematrixs=table(data_test$`Histopathologic diagnosis`,pre_svm1)
confusematrixs

## pre_svm1
## 0 1
## 0 5 3
## 1 1 19

p1=sum(diag(confusematrixs))/sum(confusematrixs)
p1

## [1] 0.8571429

#### Decision tree

# CART #
library(rpart)
library(rpart.plot)
library(maptree)

## Loading required package: cluster

rp=rpart(`Histopathologic diagnosis` ~ `ZW-FA`,data_train,method="class")
print(rp)

## n= 64
##
## node), split, n, loss, yval, (yprob)
## * denotes terminal node
##
## 1) root 64 19 1 (0.29687500 0.70312500)
## 2) ZW-FA< 54.0305 22 6 0 (0.72727273 0.27272727) *
## 3) ZW-FA>=54.0305 42 3 1 (0.07142857 0.92857143) *

summary(rp)

## Call:
## rpart(formula = `Histopathologic diagnosis` ~ `ZW-FA`, data = data_train,
## method = "class")
## n= 64
##
## CP nsplit rel error xerror xstd
## 1 0.5263158 0 1.0000000 1.0000000 0.1923709
## 2 0.0100000 1 0.4736842 0.4736842 0.1463723
##
## Variable importance
## ZW-FA
## 100
##
## Node number 1: 64 observations, complexity param=0.5263158
## predicted class=1 expected loss=0.296875 P(node) =1
## class counts: 19 45
## probabilities: 0.297 0.703
## left son=2 (22 obs) right son=3 (42 obs)
## Primary splits:
## ZW-FA < 54.0305 to the left, improve=12.42005, (0 missing)
##
## Node number 2: 22 observations
## predicted class=0 expected loss=0.2727273 P(node) =0.34375
## class counts: 16 6
## probabilities: 0.727 0.273
##
## Node number 3: 42 observations
## predicted class=1 expected loss=0.07142857 P(node) =0.65625
## class counts: 3 39
## probabilities: 0.071 0.929

rpart.plot(rp)

pre_rp=predict(rp,data_test,type="class")

### Accuracy
confusematrixt=table(data_test$`Histopathologic diagnosis`,pre_rp)
confusematrixt

## pre_rp
## 0 1
## 0 5 3
## 1 1 19

p3=sum(diag(confusematrixt))/sum(confusematrixt)
p3

## [1] 0.8571429

**Supplementary T****able 4.** Folate receptor-α expression in false-negative and false-positive samples (N = 7)

| **Number** | 1 | 2 | 3 | 4 | 5 | 6 | 7 |
| --- | --- | --- | --- | --- | --- | --- | --- |
| **Histopathologic diagnosis** | + | + | - | - | - | - | - |
| **ZW-FA diagnosis** | - | - | + | + | + | + | + |
| **immunocytochemical scoring** | 1 | 1 | 1 | 2 | 1 | 1 | 0 |
| **histological type of endometrium** | Endometrial carcinoma | Endometrial carcinoma | Atrophic endometrium | Hyperplasia endometrium | Hyperplasia endometrium | Hyperplasia endometrium | Hyperplasia endometrium |

**Supplementary Figure 1.** **^1^H NMR spectra of ZW-FA.**


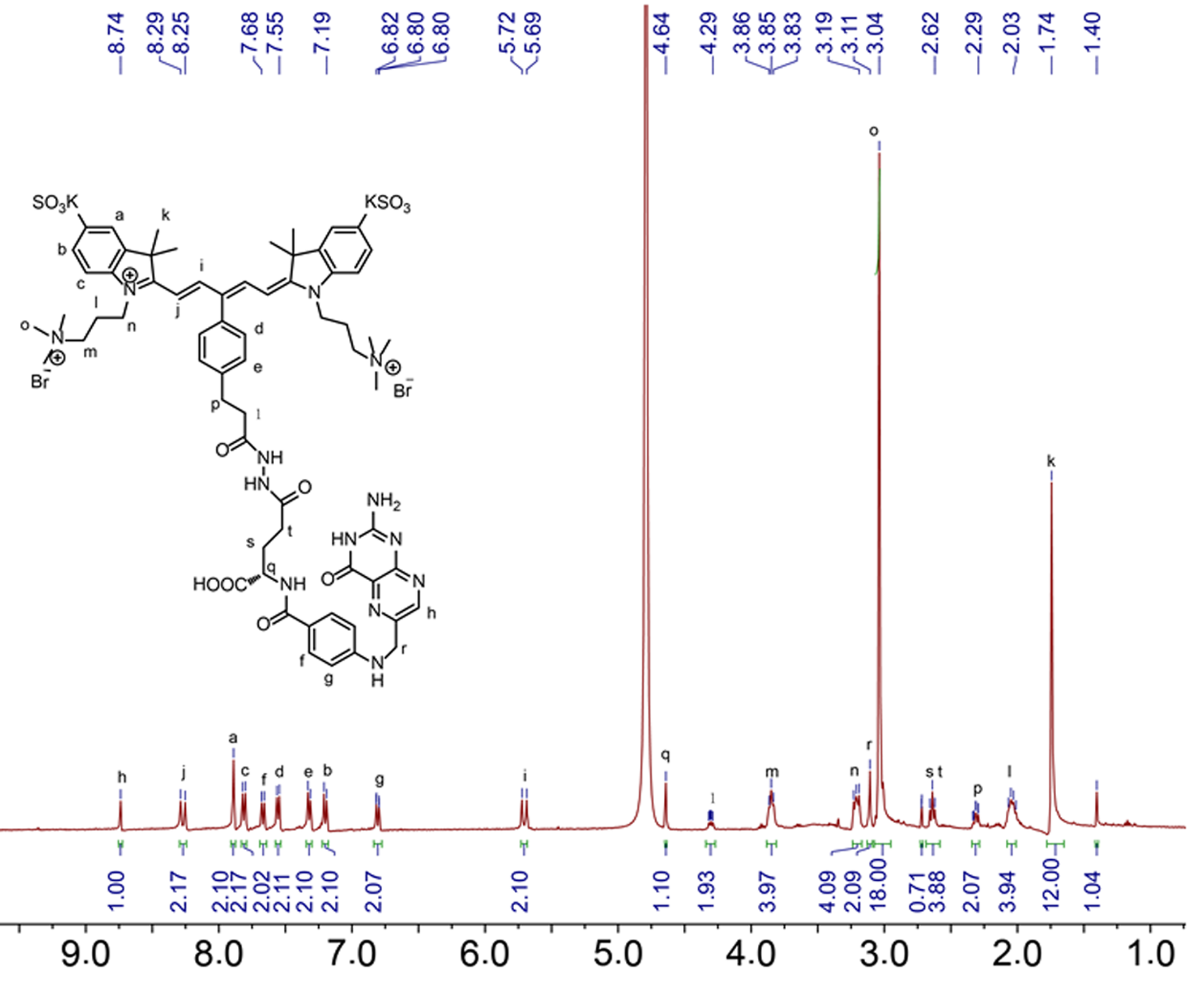

Supplement: Supplementary file 1 [file Table_1.doc]
